# Supplementary material for: The Interaction of the Endocannabinoid Anandamide and Paracannabinoid Lysophosphatidylinositol during Cell Death Induction in Human Breast Cancer Cells
Source: Int J Mol Sci. 2024 Feb 14;25(4):2271. doi: 10.3390/ijms25042271 (PMC10888638; doi:10.3390/ijms25042271)
Supplement: Supplementary file 1 [file ijms-25-02271-s001.zip › ijms-2826786-supplementary.pdf]

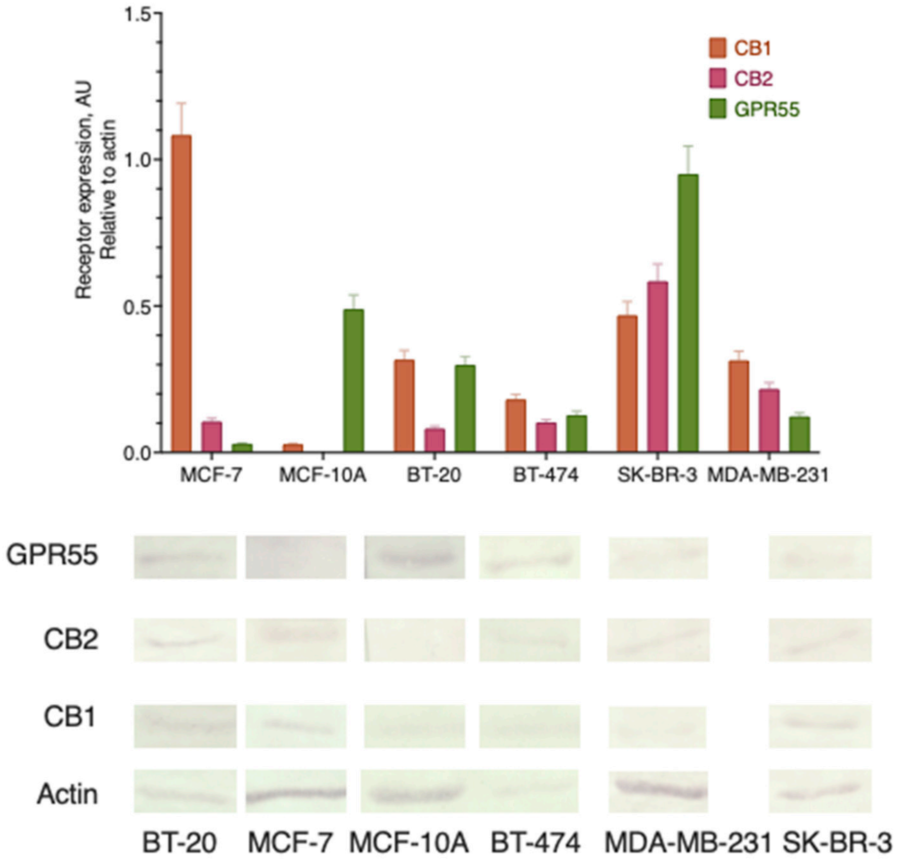

Supplement Figure S1. Western blot evaluation of the cannabinoid receptor expression in the model cell lines.
